# Effects of Dietary Hemp Harvest Residue Mixture on Carcass Characteristics, Meat Quality, and Muscle Amino Acid and Fatty Acid Profiles of Hu Ram Lambs

**DOI:** 10.3390/ani16213484

**Published:** 2026-11-04

**Authors:** Wenli Hu, Wenbin Lei, Xilong Jia, Xueyan Wang, Ting Liu, Xiaojuan Wang

**Affiliations:** College of Animal Science and Technology, Gansu Agricultural University, Lanzhou 730070, China; 18393023536@163.com (W.H.); 15294376881@163.com (W.L.); 19958584059@163.com (X.J.); 18793172652@163.com (X.W.); liuting@gsau.edu.cn (T.L.)

**Keywords:** hemp harvest residue mixture, hu lamb, meat quality, amino acids, fatty acids

## Abstract

To develop unconventional feed resources, this study investigated the effects of adding HHRM to the diet on carcass traits, meat quality, amino acids, and fatty acids in male Hu sheep lambs. Forty-eight healthy, 3-month-old lambs (24.33 ± 1.56 kg) were randomly divided into four groups (*n* = 12): CON (0%), LC (3%), MC (6%), and HC (9%). The trial included a 5-day adaptation period followed by an 80-day main trial period. Compared with the CON group, the HC group showed higher levels of GR, crude protein, EAAs, and several individual amino acids, while simultaneously reducing drip loss and improving cooking yield (*p* < 0.05). However, compared with the CON group, the HC group had a higher pH24 h value but lower b*24 h values, hardness, elasticity, cohesiveness, and chewiness; it also had the highest SFA content and the lowest PUFA, UFA, and n-6 contents. The LC and MC groups increased intramuscular fat content and reduced shear force (*p* < 0.05); among them, the MC group had the highest n-3 content (*p* < 0.05) and the lowest shear force. There were no significant differences in MUFA content. In summary, it is recommended that 6% HHRM be used as the appropriate addition level.

## Simple Summary

Fluctuations in the prices of traditional feed have prompted us to seek local, non-traditional resources. HHRM is the residue left after hemp seed harvest (seed hulls, stems, leaves, etc.) and is rich in crude protein and calcium. This study evaluated the effects of different levels of HHRM on male Hu sheep lambs. The results showed that a 9% inclusion rate increased protein and essential amino acids but reduced polyunsaturated fatty acids, whereas a 6% inclusion rate reduced meat firmness and maximized n-3 fatty acids. Therefore, 6% is the optimal inclusion rate, balancing meat quality, nutritional value, and sustainable production.

## 1. Introduction

In recent years, price fluctuations and increased supply uncertainty in conventional feed ingredients have raised the cost of sheep farming. The development of locally available, low-cost non-conventional feed resources is of great significance for maintaining dietary energy levels and reducing rearing costs [1]. Hemp and its processing by-products are rich in fiber and specific bioactive compounds, making them valuable as potential non-conventional feed resources [2]. However, research on the nutritional value of hemp, as well as its optimal inclusion levels and effects on meat quality in ruminants such as Hu sheep, remains relatively scarce and warrants further investigation.

Hemp, also known as industrial hemp, is a perennial herb with a long history of cultivation. The legal definition of industrial hemp varies by country and region; in China, the cultivation of industrial hemp with a THC content below 0.3% is legal. In recent years, global production of industrial hemp and its by-products is projected to continue growing. Hemp harvest residue mixture (HHRM) is a mixture of seed husks, stems, leaves, and stalks remaining after the harvest of mature hemp seeds, which are dried, ground, and blended in their natural proportions. According to measurements conducted by Wang Xueyan of our research group, it contains 91.60% dry matter, 3.2% crude fat, 6.65% crude fiber, 13.95% crude protein, 21.2% neutral detergent fiber, 12.6% acid detergent fiber, 24.8% crude ash, 4.21% calcium, and 0.26% phosphorus. Studies have shown that hemp seeds can increase the fat content of milk and enhance its fatty acid profile, making goat milk a high-value-added food product that is beneficial to human health [3]. Research indicates that feeding hemp seeds has no significant effect on the carcass traits of fattening sheep; however, incorporating full-fat hemp seeds into the diet has a positive effect on the fatty acid profile of beef tissue and can increase CLA content [4]. In animals fed hemp seed cake, muscle fatty acids are predominantly monounsaturated fatty acids (MUFAs), and compared with conventional protein supplements (such as soybean meal and canola meal), the MUFA proportion does not decrease significantly; in some studies, hemp seed cake has even been shown to increase the MUFA proportion [5,6]. One study found [7] that, in terms of crude protein (CP), the insoluble fraction of hemp stems (16%) was higher than that of other parts (6–8%). For hemp leaves, the immediately soluble fraction accounted for the largest proportion (20%); the data indicate that hemp leaves have nutritional and feed value for beef cattle. The addition of hemp straw to sheep diets serves as a non-conventional roughage ingredient that does not adversely affect production performance and improves nutrient digestibility [8]. In Holstein dairy cow diets, replacing alfalfa hay with a combination of industrial hemp leaf ethanol extraction byproducts and Chinese wild hay is feasible; it does not affect nutrient digestibility and can improve milk composition and plasma immune parameters, although it alters the composition of ruminal and fecal bacterial communities. This approach also has the potential to enhance economic returns from livestock production [9].

Hemp byproducts have been widely used as livestock and poultry feed worldwide. While there has been considerable research on hemp, its application in China has not yet been fully developed [10]. At present, the optimal inclusion level of HHRM in Hu sheep diets, as well as its effects on carcass traits, meat quality, and amino acid and fatty acid profiles, remain unclear. HHRM itself is not “waste” but is rich in numerous beneficial components. Incorporating hemp resources (such as stems, seeds, and leaves) into livestock production can meet the nutritional needs of livestock and poultry while achieving resource recycling, thereby yielding both economic and environmental benefits. In summary, HHRM is a highly promising unconventional feed resource, but its application in ruminant production requires further exploration. Therefore, we hypothesize that HHRM can partially replace conventional feed ingredients in Hu sheep without adversely affecting their health, and that its effects on carcass traits, meat quality, nutritional composition, and amino acid and fatty acid profiles will vary with different inclusion levels. To test this hypothesis, this study evaluated the effects of adding 0%, 3%, 6%, and 9% HHRM to the diet for 80 days on the carcass traits, meat quality, and amino acid and fatty acid profiles of male Hu sheep lambs.

## 2. Materials and Methods

### 2.1. Animals, Experimental Design, Diets, and Management

The ethical aspects of this study were approved by the Animal Ethics Committee of Gansu Agricultural University (Approval No.: GSAU-Eth-VMC-2025-051). The experimental animals were housed at the experimental base of Defu Agricultural Technology Co., Ltd. in Wuwei City, China, from April to June 2025. The HHRM used in the experiment was purchased from Minqin County, Wuwei City, China. A total of 48 healthy, three-month-old, intact male lambs of similar body weight (24.33 ± 1.56 kg) were selected for this study. They were randomly assigned to four groups (*n* = 12) based on their initial body weight: one control group and three experimental groups. Regarding the determination of the HHRM supplementation level, based on findings from the research group’s previous feeding trials, when the HHRM supplementation exceeded 9%, the lambs’ feed intake decreased sharply; therefore, the maximum supplementation level in this experiment was set at 9%. The diets for each group were as follows: Control group; LC group (3% HHRM); MC group (6% HHRM); HC group (9% HHRM). Lambs were housed individually in pens with free access to water and feed, and each animal was considered a single experimental unit. Each pen measured 23.5 m × 8.5 m and housed a total of 84 lambs, with each lamb having a living space of 1 m × 1 m; the floor was covered with slatted bamboo flooring. Each experimental group was fed hemp seed meal continuously for 85 days, including a 5-day pre-trial period and an 80-day formal trial period, with feedings administered at 8:00 a.m. and 5:00 p.m. daily. During the pre-trial period, 20% of the original feed was replaced with the experimental diet each day, and by the fifth day, the entire ration was replaced with the experimental diet. The amount of feed provided to and rejected by each lamb was weighed daily. Feeding behavior was observed in real time. The feed supply was adjusted daily to ensure a refusal rate of approximately 5% to 10%. All groups were fed a complete and balanced pelleted diet. The experimental feed formulation was developed in accordance with the standard *Nutritional Requirements for Meat Sheep* (NY/T 816-2021) [11], and nutrient analysis was conducted according to [AOAC, 2005] [12] (Table 1). The fatty acid composition of hemp residue was determined according to the GB 5009.168-2016 standard [13]; crude protein was determined according to GB/T 6432-2018 [14]; moisture content was determined according to GB/T 6435-2014; total phosphorus was determined according to GB/T 6437-2018 [15]; GB/T 6438-2007 [16] for crude ash, GB/T 6434-2022 [17] for crude fiber, GB/T 6436-2018 for calcium [18], GB/T 6433-2006 for the determination of crude fat [19]; NY/T 1459-2022 for the determination of acid-detergent fiber [20]; and GB/T 20806-2022 [21] for the determination of neutral-detergent fiber (Table 2 and Table 3). After the tests are completed, slaughter and processing shall be carried out in accordance with the *Operational Procedures for the Slaughter of Livestock and Poultry: Sheep* (GB/T 43562-2023) [22].

### 2.2. Sample Collection

On the 80th day of the formal trial period, 48 experimental sheep were first fasted for 12 h. The lambs were anesthetized by electric shock (applied to the temporal region; voltage 260 V, current 0.5 A, duration 2 s), then bled from the jugular vein and slaughtered to collect samples. Samples of the longissimus dorsi muscle were collected from the 12th to 13th intercostal spaces of the left half-carcass. Some samples were used to determine muscle pH, meat color, water loss rate, drip loss, cooked meat yield, and shear force; other samples were stored at −20 °C to determine muscle moisture, fat, and protein content; the remaining samples were placed in 1.8-mL cryotubes and stored at −80 °C for amino acid and fatty acid analysis. Carcass characteristics and meat quality were analyzed in groups of 12 lambs (*n* = 12) each; amino acids were analyzed in groups of 8 lambs (*n* = 8) each; and nutritional components and fatty acids were analyzed in groups of 6 lambs (*n* = 6) each.

### 2.3. Meat Quality Analysis

#### 2.3.1. Carcass Characteristics

After completing the standard slaughter process—including bleeding, dehairing, hoof removal, and evisceration—the whole sheep carcass was precisely cut perpendicular to the spine along a specific intercostal space (between the 5th and 6th ribs). The portion containing the forelimbs and thoracic cavity was weighed, and this weight was recorded as the forequarter weight. During slaughter, muscle samples approximately 15 cm in length and weighing approximately 160 g were collected. Prior to all analyses, visible connective tissue and fat were trimmed from the samples. In accordance with the provisions of the NY/T 2781-2015 [23] standard, marbling and external color were assessed on a cross-section of the longissimus dorsi muscle at the junction of the 12th and 13th ribs. According to [24], tissue thickness—defined as the GR value—was measured using a digital caliper (Lanzhou, China) at the junction of the 12th and 13th ribs, 11 cm from the dorsal midline; simultaneously, backfat thickness was measured using the same caliper at a point near the dorsal midline above the 12th and 13th ribs.

#### 2.3.2. Conventional Nutritional Components

The content of moisture (AOAC 950.46) [25], protein (AOAC 981.10) [26], and fat (AOAC 960.39) [27] was determined in accordance with the relevant procedures of the Association of Official Analytical Chemists (AOAC, 2005). All analyses were repeated three times.

#### 2.3.3. Basic Meat Quality Parameters

The moisture loss rate was determined using the filter paper pressing method, applying a pressure of 35 kg for 5 min with 16 layers of qualitative filter paper (Lanzhou, China) stacked on top and bottom. Moisture loss rate (%) = [(weight before pressing − weight after pressing)/weight before pressing] × 100%. A sample measuring 2.0 × 3.0 × 5.0 cm was weighed and placed in a sealed polyethylene bag; after 24 h at 4 °C, the weight was measured to calculate drip loss. Drip loss (%) = [(initial weight − final weight)/initial weight] × 100%. The sample was placed in an 80 °C water bath and heated until the center of the sample reached 70 °C. After heating, it was cooled to room temperature, the surface was blotted dry, and the sample was weighed to calculate the cooked meat yield. Cooked meat yield (%) = [(post-cooking weight/pre-cooking weight)] × 100%. Shear force was measured using a Shanghai Tengba RTA-meat muscle tenderness tester (equipped with a standard Warner-Bratzler shear blade, blade thickness 3.0 mm, internal angle 60°), in accordance with NY/T 1180-2006 [28] “Meat Tenderness Determination—Shear Force Method.” The cooked meat samples were cooled, and a 1.27 cm diameter corer was used to extract cylindrical meat columns along the direction of the muscle fibers. The meat column was placed in the blade slot so that the direction of the muscle fibers was perpendicular to the shear blade; the shear test was performed at a speed of 1.00 mm/s, and the maximum peak force (N) during the shear process was recorded. Each sample was tested three times in triplicate, and the average value was calculated. A portable pH meter (CP-461, Elmetron, Zabrze, Poland) was used with an IJ-44A probe, calibrated with pH 4.01 and 7.00 buffers. The probe was inserted into the sample to a depth of 1 cm, and three readings were taken for each sample. Meat color was measured using a Konica Minolta colorimeter (CM-2600 d, Konica Minolta, Inc., Tokyo, Japan) under a D65 light source, at a 10° observer angle, and with an 8 mm aperture. Texture characteristics were determined using a texture analyzer (TA.XT Plus, Stable Micro Systems, Godalming, Surrey, UK) under the following conditions: 50N, P/50 (50-mm-diameter aluminum cylinder); 2 × 2 × 2-cm cubes cut perpendicular to the muscle fiber direction; and a compression ratio of 50%; finally, a testing speed of 1.0 mm/s, a 2-s interval between compressions, and three technical replicates.

#### 2.3.4. Determination of Amino Acid Composition

The amino acid composition was determined in accordance with the National Food Safety Standard GB 5009.124-2016 [29]. An analytical balance (FA-1004, Shanghai Shunyu Hengping, Shanghai, China) was used to accurately weigh approximately 1.00 ± 0.10 g of the homogenized sample. Instrument conditions: Automatic amino acid analyzer (LA8080, Hitachi High-Technologies, Tokyo, Japan); detection wavelengths of 570 nm and 440 nm; injection volume of 20 μL; main pump flow rate of 0.400 mL/min; post-column reagent flow rate of 0.350 mL/min. Chromatographic column and temperature: Sulfonic acid-type cation-exchange resin column; column temperature 57 °C; post-column reaction temperature 135 ± 5 °C. Derivatization: Post-column derivatization was performed using ninhydrin solution (R1–R3). Quantification: External standard calibration was performed using a commercial amino acid standard mixture (Wako Pure Chemical Industries, Osaka, Japan). The formula for calculating content is X (g/100 g) = A × V_1_ × N/(m × V_2_ × 10,000), where A is the mass of amino acids (ng), V_1_ is the volume of hydrolysis products (mL), N is the dilution factor, m is the sample mass (g), and V_2_ is the injection volume (mL). Repeatability: Each sample was analyzed in triplicate.

#### 2.3.5. Determination of Fatty Acid Composition

The frozen meat sample was thawed at room temperature and then cut into 1-mm rectangular strips using scissors. Then, 1.00 ± 0.10 g of the meat sample was weighed and transferred to a 15-mL centrifuge tube. Next, 0.7 mL of 10 mol/L KOH (Lanzhou, China) and 5.3 mL of anhydrous methanol (Lanzhou, China) were added to the centrifuge tube, which was maintained in a 55 °C water bath for 1.5 h and shaken for 5 s every 20 min. After the water bath, the centrifuge tube was cooled to room temperature under running tap water. Next, 0.58 mL of 12 mol/L H_2_SO_4_ (Lanzhou, China) was added to the cooled centrifuge tube, inverted and mixed, and the first water bath process was repeated. After cooling, 1 mL of n-hexane (Lanzhou, China) was added to the centrifuge tube and shaken well. The mixture was centrifuged at 1110× *g* for 5 min, and then the supernatant was collected. The supernatant was filtered through a sterile syringe (Lanzhou, China) into a brown bottle and stored at −20 °C for analysis. Lipids were extracted from meat samples via the methylation of fatty acids (FAs) using the method described by O’Fallon et al. [30]. The composition and content of FAs in LD muscle samples were analyzed using an Agilent 6890N gas chromatograph (Agilent Technologies, Inc., Santa Clara, CA, USA) based on peak area and retention time. GC analysis conditions: SP-2560 column (100 m × 0.25 mm × 0.2 μm, Sigma-Aldrich, St. Louis, MO, USA); initial temperature of 140 °C held for 5 min, then ramped at 2 °C/min to 200 °C and held for 5 min, followed by a ramp at 6 °C/min to 230 °C and held for 20 min; detector temperature: 250 °C; injector temperature: 220 °C; nitrogen flow rate: 1.2 mL/min; split ratio: 100:1; injection volume: 1 μL; carrier gas: high-purity nitrogen. The content of individual fatty acids was quantified based on peak area and expressed as a percentage of total fatty acids. The internal standard was methyl heneicosanoate (Solarbio, Beijing, China, SKU SM5940-100 mg), with a purity of ≥98%.

#### 2.3.6. Data Analysis

Data were organized using Excel 2003 (Microsoft Corp., Redmond, WA, USA), and one-way analysis of variance (ANOVA) was performed using SPSS 27.0 software (IBM Corp., Armonk, NY, USA) to test for significant differences between groups. The significance level for all tests was set at *p* < 0.05, followed by Tukey’s HSD test for multiple comparisons. SEM: standard error of the mean.

## 3. Results

### 3.1. Effects of HHRM on Carcass Traits

As shown in Table 4, the GR value in the HC group was significantly higher than those in the CON and MC groups (*p* < 0.05), but the difference compared with the LC group was not significant (*p* > 0.05). The GR value in the LC group was significantly higher than that in the CON group (*p* < 0.05), while the differences between the MC group and the CON group, and between the LC group and the MC group, were not significant (*p* > 0.05). None of the treatment levels had a significant effect on backfat thickness, meat color score, marbling score, or forequarter weight (*p* > 0.05).

### 3.2. Effects of HHRM on the Nutritional Composition

As shown in Table 5, crude fat content was significantly higher in both the LC and MC groups than in the CON group (*p* < 0.05); there were no significant differences between the LC and MC groups, or between the HC group and the other three treatment groups (*p* > 0.05). Protein content in the HC group was significantly higher than in the CON, LC, and MC groups (*p*< 0.05). The different treatment levels had no significant effect on moisture content (*p* > 0.05).

### 3.3. Effects of HHRM on the Meat Quality Parameters

As shown in Table 6, drip loss in the CON group was significantly higher than in the LC, MC, and HC groups (*p* < 0.05); the HC group was significantly lower than the LC group (*p* < 0.05), with no significant difference from the MC group (*p* > 0.05). The cooked meat yield in the HC group was significantly higher than that in the CON group (*p* < 0.05), with no significant differences observed between the HC group and either the LC or MC groups (*p* > 0.05). Shear force in the CON group was significantly higher than in the LC, MC, and HC groups (*p* < 0.05), with no significant differences among the treatment groups (*p* > 0.05). At pH 45 min, the CON group was significantly higher than the LC and HC groups (*p*< 0.05), with no significant differences among the treatment groups. At 24 h, the pH values in the LC and HC groups were significantly higher than those in the CON group (*p* < 0.05), while there were no significant differences between the MC group and the CON, LC, or HC groups (*p* > 0.05). For b* at 24 h, the values in the CON and LC groups were significantly higher than those in the MC and HC groups (*p* < 0.05). Cohesiveness in the CON group was significantly higher than in the LC, MC, and HC groups; the LC group was significantly higher than the HC group (*p* < 0.05); and there were no significant differences between the LC and MC groups or between the MC and HC groups (*p* > 0.05). Gumminess in the LC group was significantly higher than in the CON, MC, and HC groups; the CON group was significantly higher than the MC and HC groups (*p* < 0.05); and there were no significant differences between the MC and HC groups. The springiness of the CON group was significantly higher than that of the LC, MC, and HC groups (*p* < 0.05), and there were no significant differences among the experimental groups. The chewability of the CON group was significantly higher than that of the LC, MC, and HC groups; the LC group was significantly higher than the MC and HC groups (*p* < 0.05), and there were no significant differences between the MC and HC groups. Hardness in the LC group was significantly higher than in the CON, MC, and HC groups (*p* < 0.05); the CON group was significantly higher than the HC group; and there were no significant differences between the MC group and either the CON or HC groups. Different treatment levels had no significant effect on water loss rate, meat color at 45 min, L*24 h, or a*24 h (*p* > 0.05).

### 3.4. Effects of HHRM on the Amino Acid Composition

As shown in Table 7, Thr, Met, His, Asp, Ser, Glu, Gly, Ala, Cys, Pro, NEAA, TAA, UAA, and SAA did not differ significantly among the groups (*p* > 0.05). Val and Phe levels in the HC group were significantly higher than those in the CON, LC, and MC groups (*p* < 0.05); no significant differences were observed among the other three groups. Ile levels in the HC group were significantly higher than those in the CON group (*p* < 0.05); the LC and MC groups showed no significant differences compared with either the CON or HC groups (*p* > 0.05). Leu, Lys, Arg, and Tyr levels in the HC group were significantly higher than those in the CON and LC groups (*p* < 0.05); the MC group showed no significant differences compared with the other three groups (*p* > 0.05). For Phe, the HC group was significantly higher than the CON, LC, and MC groups (*p* < 0.05). The total EAAs in the HC group were significantly higher than those in the CON and LC groups (*p* < 0.05); the MC group showed no significant differences from the other groups (*p* > 0.05). The EAAs/TAA in the HC group were significantly higher than those in the CON and MC groups (*p* < 0.05); the LC group showed no significant differences from the other groups (*p* > 0.05). The levels of BTAA in the HC group were significantly higher than those in the CON and LC groups (*p* < 0.05); the MC group showed no significant differences from the other groups (*p* > 0.05).

### 3.5. Effects of HHRM on the Fatty Acid Composition

As shown in Table 8, for C4, C10, C11, C12, C13, C14, C24, C15:1, C16:1, C17:1, C18:1T, C18:1 n-9, C22:1 n-9, C24:1 n-9, C18:2T, C18:2 n-6, C18:3 n-3, C20:4 n-6, C22:2 n-6, C20:5 n-3, C22:6 n-3 and MUFA—there were no significant differences among the groups (*p* > 0.05). For C6, the LC and MC groups were significantly higher than the HC group (*p* < 0.05); the CON group showed no significant differences compared with the other three groups (*p* > 0.05). For C8, the MC group was significantly higher than the CON and HC groups (*p* < 0.05). For C15, the MC group was significantly higher than the CON and LC groups (*p* < 0.05). For C16, the MC group was significantly higher than the CON, LC, and HC groups (*p* < 0.05), and the HC group was significantly higher than the CON group (*p* < 0.05). For C17 and C18, there were no significant differences among the CON, LC, and HC groups (*p* > 0.05), but all were significantly higher than the MC group (*p* < 0.05). C20: The levels in the CON and LC groups were significantly higher than those in the MC and HC groups (*p* < 0.05). C22: The level in the MC group was significantly higher than that in the CON, LC, and HC groups (*p* < 0.05). C23: The level in the HC group was significantly lower than that in the other three groups (*p* < 0.05); there were no significant differences among the other three groups (*p* > 0.05). C14:1: The levels in the CON and LC groups were significantly higher than those in the MC and HC groups (*p* < 0.05). C18:3 n-6: The level in the MC group was significantly higher than that in the HC group (*p* < 0.05). C20:1: The level in the MC group was significantly lower than that in the other three groups (*p* < 0.05); there were no significant differences among the other three groups (*p* > 0.05). C20:2 n-6: The level in the MC group was significantly higher than that in the CON, LC, and HC groups (*p* < 0.05). C20:3 n-6 levels were significantly higher in the CON and LC groups than in the HC and MC groups (*p* < 0.05), and the HC group was significantly higher than the MC group (*p* < 0.05). C20:3 n-3 levels were significantly higher in the MC group than in the other three groups (*p* < 0.05). Total n-3 fatty acids in the MC group were significantly higher than in the other three groups; the HC group was significantly higher than the CON and LC groups; and there was no significant difference between the CON and LC groups. Total n-6 fatty acids, PUFA, and UFA in the HC group were significantly lower than those in the CON, LC, and MC groups (*p* < 0.05). SFAs were significantly higher in the HC group than in the CON, LC, and MC groups (*p* < 0.05).

## 4. Discussion

In recent years, nutritional regulation has become a key strategy for enhancing the nutritional value of meat products, and changes in fat and crude protein content in muscle are closely related to its nutritional characteristics [31]. The GR value is a core indicator for evaluating fat deposition levels in sheep. Previous studies have shown that feeding hemp by-products leads to changes in amino acid and lipid metabolism in lambs, and that cholesterol synthesis in lambs is generally activated [32]. This is consistent with the macroscopic phenotypic results of this study: in this study, the GR value of the 9% (HC) group was significantly higher than those in the CON and MC groups, which may be due to the higher dose of HHRM promoting trunk fat accumulation by influencing lipid metabolic pathways. However, this study did not quantitatively determine the lipophilic bioactive compounds in HHRM, and further verification is needed. Intramuscular fat content is highly correlated with the sensory quality of lamb meat and plays an important role in tenderness, juiciness, and flavor [33]; intramuscular fat content is inversely proportional to muscle water content. In this study, the crude fat content in the LC and MC groups was significantly higher than that in the control group, while there was no significant difference between the HC group and the control group; conversely, the crude protein content in the HC group was significantly higher than that in the other three groups. Since the HC group was numerically the lowest among the four groups, the increase in protein on a fresh meat basis was partly attributable to the “concentration effect.” When converted to a dry matter basis, the protein level in the HC group was nearly equal to that in the LC group.

Meat quality is typically evaluated using indicators such as drip loss, cooking yield (or cooking loss), and shear force; among these, cooking loss is a commonly used indicator of muscle water-holding capacity [34]. This study found that the addition of HHRM significantly reduced drip loss and shear force in muscle tissue. Muscle pH is determined by various factors, including H^+^ released from the hydrolysis of ATP, the accumulation of lactic acid produced by glycolysis, the breakdown of creatine phosphate, and the intrinsic buffering capacity of muscle proteins [35]. In the LC and HC groups, pH decreased 45 min post-slaughter, thereby avoiding the risk of DFD (dark, firm, dry) meat associated with high pH; however, the pH decline over 24 h was significantly lower than that in the control group. The higher final pH value shifted myofibrillar proteins away from their isoelectric points, widening the gaps between myofibrils through electrostatic repulsion, thereby enhancing the muscle’s water-holding capacity. This was directly reflected in reduced drip loss and improved cooking yield [36]. Furthermore, a high pH environment helps maintain the activity of post-slaughter calcium-activated enzymes (calpains), promoting the degradation of myofibrillar proteins [37], which explains the significantly reduced shear force observed in the HHRM groups. However, a high final pH is a double-edged sword. When the pH of lamb meat approaches 6.20 at 24 h, it typically exhibits characteristics similar to DFD meat. Although high pH improves water-holding capacity and tenderness, the b* values at 24 h in the MC and HC groups in this study were significantly reduced, which is consistent with the slowed rate of myoglobin oxidation under high pH conditions and the deeper, dark red color of the meat. It should be noted that dark-colored meat that has not undergone oxidation does not necessarily indicate improved appearance. On the contrary, meat with a high pH is typically associated with an increased risk of microbial growth, which is not conducive to extending the commercial shelf life. Meat quality assessment can also be aided by texture profile analysis (TPA). This study indicates that the addition of HHRM to the diet significantly reduced the cohesiveness and chewiness of muscle tissue. Lower cohesiveness and chewiness may reflect a looser muscle microstructure or reduced connective tissue cross-linking [38,39]. In summary, the effects of adding HHRM to the diet on the physical properties of lamb meat are primarily achieved by altering the post-slaughter glycolytic process. Specific fiber levels and fatty acid composition in HHRM may have influenced ruminal fermentation patterns and muscle glycogen reserves, thereby regulating the rate of post-slaughter pH decline; however, this hypothesis requires confirmation through future metabolomics and molecular mechanism studies.

The composition and content of amino acids not only determine the nutritional value of meat but are also closely related to its flavor complexity, tenderness, and oxidative stability. Previous studies have confirmed that Glu and Asp are the key compounds responsible for the umami flavor in meat, while Lys is a key precursor involved in the Maillard reaction during heating that produces volatile meat aromas [40]. In this study, the Lys, Phe, and Tyr contents in the HC group (9% addition rate) were significantly higher than those in the control group, while Glu and Asp showed a numerically increasing trend. These results indicate that a 9% supplementation of HHRM can effectively improve amino acid deposition in muscle, which may be related to the amino acid composition of HHRM itself and its degradation kinetics in the rumen, thereby providing the muscle with a richer supply of amino acid precursors. Branched-chain amino acids (BCAAs, including leucine, isoleucine, and valine) are not only important substrates for muscle protein synthesis but also act as key signaling molecules involved in metabolic regulation [41,42]. In this study, the Ile, Val, and Leu contents, as well as the EAA, in the HC group were significantly higher than in the other groups. As a non-conventional feed with low NDF and relatively high CP, the addition of HHRM may have altered the fermentation patterns of rumen microorganisms. Studies in Hu sheep further indicate that changes in rumen microbial function can enhance the biosynthesis of amino acids such as branched-chain amino acids and promote microbial protein production [43]. At the same time, hemp cake meal is a good source of rumen-undegradable protein (RUP), which escapes ruminal degradation and releases amino acids in the small intestine for absorption [44]. Taken together, these findings suggest that the addition of HHRM may increase the supply of high-quality bypass amino acids (such as BCAAs) to the small intestine by regulating ruminal fermentation and microbial protein synthesis, thereby promoting the deposition of EAAs in muscle. Since this study used acid hydrolysis to determine total amino acids (bound + free), the aforementioned changes in Glu and Asp levels cannot be directly equated with an increase in free umami compounds or a direct improvement in meat flavor. Furthermore, although the improvement in the amino acid profile suggests enhanced protein anabolism, this study did not measure the gene expression of relevant signaling pathways in muscle. Future research should combine free amino acid-targeted metabolomics with molecular biological approaches to comprehensively evaluate the regulatory mechanisms of HHRM on lamb flavor and protein metabolism.

The fatty acid composition of meat not only affects meat quality and flavor but is also closely related to human dietary health [45]. Odd-chain fatty acids (such as C15:0 and C17:0) are primarily derived from propionic acid produced by rumen microbial fermentation [46]. The results of this study indicate that the addition of HHRM to the diet had a significant effect on the deposition of odd-chain fatty acids in muscle: the MC group hadthe highest C15:0 content in numerical terms and significantly the lowest C17:0 content. The fiber and polyunsaturated fatty acids in HHRM may have altered the ruminal environment, thereby reshaping the structure of propionic acid-producing and hydrogen-producing microbial communities, leading to a redistribution of VFA absorption patterns and microbial precursor synthesis [47]. However, C15:0 and C17:0 exhibited opposite deposition trends; this suggests that the elongation or oxidation metabolic pathways of odd-chain fatty acids with different carbon chain lengths may differ in tissues. In this study, although the HHRM feed was rich in C18:1 n-9, C18:2n-6 and C18:3n-3, there were no significant differences in the levels of C18:1 n-9, C18:2n-6 and C18:3n-3 among the groups. This is primarily attributed to the powerful biological hydrogenation in the rumen of ruminants; the vast majority of dietary PUFAs are reduced to SFAs in the rumen, and only a small amount escapes ruminal degradation to enter the small intestine [48]. From a new perspective on the endogenous regulation of lipid metabolism, this study found that the MC group had a numerically very high proportion of MUFA and C18:1 n-9. This is typically associated with high activity of stearoyl-CoA desaturase (SCD), which is responsible for converting C18:0 to C18:1 [49]. The supply of specific fatty acid substrates in the diet may have upregulated the expression of the SCD gene in muscle. The C18:0 levels in the MC group were extremely low, resulting in a numerical decrease in its SFA. From a biological mechanism perspective, the extremely high SCD enzyme activity in the MC group may rapidly convert large amounts of C18:0 into C18:1 at the tissue level; However, this hypothesis requires further confirmation through SCD enzyme activity assays and optimized chromatographic separation conditions. In summary, the addition of HHRM to the diet can influence the deposition of specific fatty acids by regulating ruminal microbial fermentation patterns; however, its effect on the overall SFA-to-MUFA ratio in muscle is constrained by both analytical methods and metabolic pathways. Future research should integrate lipidomics and molecular biology approaches to precisely elucidate the underlying mechanisms by which HHRM affects lipid metabolism in sheep.

## 5. Conclusions

The results showed that, compared with the CON group, the HC group had higher levels of GR, crude protein, EAA, and amino acids such as Val, Ile, Leu, Phe, and Lys, while also exhibiting lower drip loss and higher cooking yield (*p* < 0.05). However, compared with the CON group, the HC group had a higher pH24 h value but lower b*24 h values, hardness, elasticity, cohesiveness, and chewiness; it also had the highest SFA content and the lowest PUFA, UFA, and n-6 fatty acid contents, indicating that its quality advantages were selective rather than comprehensive. The LC and MC groups increased intramuscular fat content and reduced shear force (*p* < 0.05); among them, the MC group had the highest n-3 fatty acid content and the lowest shear force value. There was no significant difference in MUFA content. The pH45min and pH24 h values should be interpreted separately; the higher pH24 h value indicates a reduced degree of post-slaughter acidification rather than an improvement in quality. In summary, a 6% HHRM supplementation level exhibited a relatively balanced response and is recommended as the more appropriate addition level.

## 6. Limitations of the Study

This study has the following limitations: First, no quantitative analysis was conducted on bioactive components, such as cannabinoids, in HHRM, nor was the antioxidant status in the animals assessed. Second, the acid hydrolysis method used in this study measured total amino acids, which does not fully represent the actual flavor contribution of free amino acids in muscle tissue; furthermore, no sensory evaluation of the meat products was conducted, and changes in texture profile analysis (TPA) parameters only reflect alterations in physical properties and cannot be equated with improvements in consumer palatability. Third, in the gas chromatographic analysis of fatty acids, co-elution and abnormal integration were observed for the C18:0 and C18:1 peaks in some samples (e.g., abnormally low C18:0 data in the MC group), which somewhat affected the accuracy of the total saturated and monounsaturated fatty acid measurements. Finally, this study did not measure rumen fermentation parameters, the activity of relevant desaturase enzymes (such as SCD), or gene expression levels; therefore, the underlying molecular mechanisms remain speculative. Future research should optimize chromatographic separation conditions and integrate free amino acid metabolomics, sensory evaluation, and molecular biology approaches to comprehensively elucidate the underlying mechanisms by which hemp seed meal regulates lamb meat quality.

## Figures and Tables

**Table 1:** Feed composition and nutritional levels.

Items	CON	LC	MC	HC
Corn stover	20.00	17.87	16.89	15.4
Maize	33.80	32.00	30.65	29.2
Corn husk	11.00	12.90	13.00	13.10
Cottonseed meal	5.00	5.00	5.00	5.00
Soybean meal	5.50	4.50	3.50	2.50
Corn germ meal	18.00	18.20	18.40	19.20
HHRM	0.00	3.00	6.00	9.00
Expanded urea	0.50	0.53	0.56	0.60
Limestone	0.90	0.70	0.70	0.70
Molasses	1.50	1.50	1.50	1.50
NaHCO_3_	1.00	1.00	1.00	1.00
NaCl	0.70	0.70	0.70	0.70
Premix ^1^	2.10	2.10	2.10	2.10
Total	100.00	100.00	100.00	100.00
Nutrition level				
Metabolic energy, MJ/Kg ^2^	9.75	9.56	9.57	9.44
Dry matter,%	91.68	91.69	92.28	92.07
Crude protein,%	14.40	14.49	14.67	14.12
Neutral detergent fiber,%	39.79	41.55	41.47	42.64
Acid detergent fiber,%	14.21	14.50	14.98	16.04
Crude ash,%	9.14	11.29	10.64	11.32

^1^ The premix provided the following per kg of diets: VA 3 100 IU, VD3 1 300 IU, Fe 24 mg, Cu 8 mg, Co 0.1 mg, Mn 30 mg, Se 0.35 mg, Zn 30 mg, I 0.45 mg. ^2^ ME was a calculated value, while the others were measured values.

**Table 2:** Nutritional level of HHRM.

Nutrition Level (%)			
Dry matter	91.60	Crude ash	24.80
Crude fat	3.20	Calcium	4.21
Crude protein	13.95	Total phosphorus	0.26
Neutral detergent fiber	21.20	Crude fiber	6.60
Acid detergent fiber	12.60		

**Table 3:** Fatty acid composition of HHRM.

Fatty Acid (g/100 g)			
C4:0	0.0032	C18:2n6c	0.6700
C6:0	0.0084	C20:0	0.0671
C8:0	0.0015	C18:3n6	0.0112
C10:0	0.0033	C20:1	0.7450
C12:0	0.0268	C18:3n3	0.0078
C13:0	0.0138	C21:0	0.0076
C14:0	0.0646	C20:2	0.0039
C14:1	0.0091	C22:0	0.0689
C15:0	0.0032	C20:3n6	0.0018
C16:0	0.5280	C20:3n3	0.0030
C16:1	0.0172	C20:4n6	0.0020
C17:0	0.0077	C22:1n9	0.0033
C17:1	0.0018	C23:0	0.0139
C18:0	0.0994	C20:5n3	0.0051
C18:1n9t	0.0012	C24:0	0.0667
C18:1n9c	0.2210	C24:1	0.0020
C18:2n6t	0.0043		

**Table 4:** Effects of HHRM on carcass traits.

Items	CON	LC	MC	HC	SEM	*p* Value
Back fat thickness, mm	5.80	5.90	6.10	6.40	0.023	0.824
Meat color score	3.67	3.55	3.50	3.44	0.072	0.776
Marbling	3.35	3.54	3.44	3.38	0.054	0.559
GR, mm	19.20 ^c^	23.80 ^ab^	21.80 ^bc^	26.40 ^a^	0.064	<0.001
Forequarter weight, kg	7.17	7.25	7.23	7.27	0.095	0.984

^a–c^ indicate significant differences between means (*p* < 0.05). Protein content in the HC group was significantly higher than in the CON, LC, and MC groups (*p* < 0.05). The different treatment levels had no significant effect on moisture content (*p* > 0.05). The same applies below.

**Table 5:** Effects of HHRM on the nutritional composition.

Items	CON	LC	MC	HC	SEM	*p* Value
Moisture, %	72.39	73.59	72.42	70.94	0.366	0.066
Crude fat, %	2.67 ^b^	3.43 ^a^	3.47 ^a^	3.09 ^ab^	0.108	0.021
Protein, %	21.98 ^b^	22.02 ^b^	22.56 ^b^	24.26 ^a^	0.198	<0.001

**Table 6:** Effects of HHRM on basic meat quality parameters.

Items	CON	LC	MC	HC	SEM	*p* Value
Pressure-induced water loss, %	26.85	25.22	26.26	24.52	0.408	0.209
Drip loss, %	4.25 ^a^	3.21 ^b^	2.95 ^bc^	2.76 ^c^	0.117	<0.001
Cooking yield, %	53.65 ^b^	55.70 ^ab^	54.24 ^ab^	56.38 ^a^	0.388	0.048
Shear force, N	24.12 ^a^	19.95 ^b^	17.80 ^b^	19.27 ^b^	0.731	0.013
pH value						
45 min	6.86 ^a^	6.65 ^b^	6.73 ^ab^	6.70 ^b^	0.027	0.030
24 h	5.87 ^b^	6.20 ^a^	6.00 ^ab^	6.21 ^a^	0.045	0.020
Meat color						
L*45 min	35.08	35.65	34.95	34.54	0.203	0.263
a*45 min	16.76	16.87	16.75	16.81	0.183	0.995
b*45 min	5.84	5.99	5.82	5.43	0.087	0.106
L*24 h	37.80	37.68	37.07	36.65	0.399	0.730
a*24 h	19.62	18.06	18.86	17.96	0.291	0.168
b*24 h	12.13 ^a^	11.57 ^a^	9.64 ^b^	10.18 ^b^	0.283	<0.001
Cohesiveness	0.46 ^a^	0.39 ^b^	0.36 ^bc^	0.32 ^c^	0.009	<0.001
Gumminess, kgf	1.25 ^b^	1.85 ^a^	0.71 ^c^	0.41 ^c^	0.101	<0.001
Springiness, mm	2.12 ^a^	0.70 ^b^	0.77 ^b^	0.73 ^b^	0.143	<0.001
Chewiness, N·mm	2.89 ^a^	1.03 ^b^	0.65 ^c^	0.41 ^c^	0.205	<0.001
Hardness, N	27.16 ^b^	45.41 ^a^	18.93 ^bc^	12.06 ^c^	2.160	<0.001

**Table 7:** Effects of HHRM on the amino acid composition. (g/100 g).

Items	CON	LC	MC	HC	SEM	*p* Value
EAA						
Threonine (Thr)	0.82	0.82	0.83	0.89	0.010	0.064
Valine (Val)	1.02 ^b^	1.03 ^b^	1.02 ^b^	1.09 ^a^	0.010	0.017
Methionine	0.30	0.28	0.29	0.32	0.006	0.250
Isoleucine (Ile)	0.88 ^b^	0.90 ^ab^	0.91 ^ab^	0.97 ^a^	0.012	0.022
Leucine (Leu)	1.47 ^b^	1.49 ^b^	1.52 ^ab^	1.62 ^a^	0.020	0.022
Phenylalanine (Phe)	0.78 ^b^	0.80 ^b^	0.83 ^b^	0.90 ^a^	0.013	<0.001
Lysine (Lys)	1.66 ^b^	1.68 ^b^	1.70 ^ab^	1.81 ^a^	0.021	0.037
Histidine (His)	0.60	0.60	0.63	0.65	0.009	0.118
Arginine (Arg)	1.16 ^b^	1.17 ^b^	1.19 ^ab^	1.26 ^a^	0.015	0.048
NEAA						
Aspartate (Asp)	1.61	1.63	1.65	1.73	0.020	0.096
Serine (Ser)	0.62	0.60	0.64	0.66	0.008	0.084
Glutamate (Glu)	2.54	2.57	2.61	2.77	0.035	0.079
Glycine (Gly)	0.79	0.79	0.81	0.83	0.009	0.384
Alanine (Ala)	1.03	1.03	1.05	1.09	0.012	0.174
Cystine (Cys)	0.13	0.15	0.13	0.14	0.004	0.245
Tyrosine (Tyr)	0.66 ^b^	0.64 ^b^	0.70 ^ab^	0.73 ^a^	0.012	0.028
Proline (Pro)	0.69	0.70	0.71	0.71	0.008	0.690
EAA	8.66 ^b^	8.74 ^b^	8.91 ^ab^	9.47 ^a^	0.112	0.027
NEAA	8.06	8.10	8.30	8.66	0.100	0.113
TAA	16.72	16.84	17.21	18.13	0.211	0.054
EAA/TAA	51.77 ^b^	51.90 ^ab^	51.78 ^b^	52.26 ^a^	0.068	0.043
UAA	4.15	4.19	4.26	4.50	0.054	0.082
SAA	5.60	5.62	5.74	5.98	0.065	0.124
BTAA	6.96 ^b^	7.02 ^b^	7.20 ^ab^	7.65 ^a^	0.094	0.025

Note: EAA, essential amino acids (Thr + Val + Met + Ile + Leu + Phe + Lys + His + Arg); NEAA, non-essential amino acids (Asp + Ser + Glu + Gly + Ala + Cys + Tyr + Pro); TAA, total amino acids (EAA + NEAA); UAA, umami amino acids (Asp + Glu); SAA, sweet amino acids (Thr + Ser + Gly + Ala + Pro + Lys); BTAA, bitter amino acids (Val + Met + Ile + Leu + Tyr + Phe + His + Arg + Cys).

**Table 8:** Effects of HHRM on the fatty acid composition. (% of total fatty acids).

Fatty Acid	CON	LC	MC	HC	SEM	*p* Value
C4	0.0032	0.0034	0.0047	0.0027	0.001	0.544
C8	0.0071 ^b^	0.0095 ^ab^	0.0122 ^a^	0.0069 ^b^	0.001	0.004
C10	0.1104	0.1330	0.1570	0.1158	0.010	0.228
C11	0.0094	0.0081	0.0114	0.0035	0.001	0.194
C12	0.0584	0.0645	0.0762	0.0698	0.004	0.359
C13	0.2791	0.3468	0.3156	0.0311	0.048	0.089
C14	1.6477	1.8458	2.3066	2.1453	0.123	0.198
C14:1	0.3415 ^a^	0.4081 ^a^	0.1117 ^b^	0.0990 ^b^	0.043	0.001
C15	0.2143 ^b^	0.2256 ^b^	0.3154 ^a^	0.2761 ^ab^	0.015	0.034
C15:1	0.2382	0.2512	0.2649	0.0711	0.032	0.112
C16	22.6596 ^c^	23.9020 ^bc^	29.0192 ^a^	25.4364 ^b^	0.705	<0.001
C16:1	1.8968	2.0825	2.0757	1.7799	0.103	0.727
C17	0.9503 ^a^	0.8600 ^a^	0.2806 ^b^	1.0397 ^a^	0.087	<0.001
C17:1	0.7615	0.7400	0.8345	0.6365	0.032	0.124
C18	12.6557 ^a^	12.3758 ^a^	0.0580 ^b^	13.7782 ^a^	1.599	<0.001
C18:1T	4.1896	3.3616	3.4205	5.1248	0.348	0.254
C18:1 n-9	40.4142	39.1552	42.0770	39.9390	0.653	0.500
C18:2T	0.1615	0.1388	0.1230	0.1558	0.008	0.250
C18:2 n-6	8.3498	8.2763	7.6560	6.4838	0.448	0.536
C20	0.0396 ^a^	0.0375 ^a^	0.0141 ^b^	0.0187 ^b^	0.003	<0.001
C18:3 n-6	0.0783 ^ab^	0.0736 ^ab^	0.1132 ^a^	0.0644 ^b^	0.007	0.032
C20:1	0.1138 ^a^	0.1080 ^a^	0.0336 ^b^	0.1019 ^a^	0.009	<0.001
C18:3 n-3	0.3164	0.3060	0.3468	0.3668	0.019	0.744
C20:2 n-6	0.1566 ^b^	0.1204 ^b^	0.4219 ^a^	0.0973 ^b^	0.038	<0.001
C22	0.0830 ^b^	0.0650 ^b^	0.1477 ^a^	0.0384 ^b^	0.013	<0.001
C20:3 n-6	0.2767 ^a^	0.3044 ^a^	0.0622 ^c^	0.1469 ^b^	0.027	<0.001
C20:3 n-3	0.0026 ^b^	0.0045 ^b^	0.2391 ^a^	0.0025 ^b^	0.032	<0.001
C22:1 n-9	0.0173	0.0176	0.0158	0.0064	0.002	0.103
C20:4 n-6	2.4550	3.8779	3.3349	1.5448	0.461	0.388
C23	0.0716 ^a^	0.0867 ^a^	0.1052 ^a^	0.0158 ^b^	0.010	0.001
C22:2 n-6	0.0580	0.0486	0.0449	0.0369	0.008	0.868
C24	0.0151	0.0313	0.0133	0.0166	0.003	0.109
C20:5 n-3	0.0144	0.0113	0.0299	0.0056	0.006	0.481
C24:1 n-9	0.5833	0.6566	0.5393	0.3184	0.058	0.289
C22:6 n-3	0.0539	0.0470	0.0451	0.0502	0.006	0.956
n3	0.3873 ^c^	0.3700 ^c^	0.6572 ^a^	0.4346 ^b^	0.033	<0.001
n6	11.3725 ^a^	12.6922 ^a^	11.6324 ^a^	8.3663 ^b^	0.539	0.005
SFA	39.5037 ^b^	39.9949 ^b^	38.2195 ^b^	42.9920 ^a^	0.584	0.004
MUFA	48.5561	46.7808	49.3697	48.0694	0.432	0.265
PUFA	11.9286 ^a^	13.2088 ^a^	12.4124 ^a^	8.9596 ^b^	0.530	0.007
UFA	60.4805 ^a^	59.9896 ^a^	61.7863 ^a^	57.0006 ^b^	0.619	0.006

Saturated fatty acids (SFAs); Monounsaturated fatty acids (MUFA); Polyunsaturated fatty acids (PUFAs); Unsaturated fatty acids (UFA); Total unsaturated fatty acids (TUFA). SFA = sum of C4:0, C8:0, C10:0, C11:0, C12:0, C13:0, C14:0, C15:0, C16:0, C17:0, C18:0, C20:0, C22:0, C23:0, and C24:0.; MUFA = sum of C14:1, C15:1, C16:1, C17:1, C18:1T, C18:1 n-9, C20:1, C22:1 n-9, and C24:1 n-9. PUFA = sum of C18:2T, C18:2 n-6, C18:3 n-6, C18:3 n-3, C20:2 n-6, C20:3 n-6, C20:3 n-3, C20:4 n-6, C22:2 n-6, C20:5 n-3, and C22:6 n-3. n-3 = sum of C18:3 n-3, C20:3 n-3, C20:5 n-3, and C22:6 n-3. n-6 = sum of C18:2 n-6, C18:3 n-6, C20:2 n-6, C20:3 n-6, C20:4 n-6, and C22:2 n-6.

## Data Availability

All data in this study are available from the authors upon reasonable request.

## Abbreviations

The following abbreviations are used in this manuscript:

BCAA	Branched-chain amino acids
BTAA	Bitter-tasting amino acids
DFD	Dark, firm, dry
EAA	Essential amino acids
HHRM	Hemp harvest residue mixture
MUFA	Monounsaturated fatty acids
NEAA	Nonessential amino acids
PUFA	Polyunsaturated fatty acids
RUP	Rumen-undegradable protein
SAA	Sweet amino acids
SCD	Stearoyl-CoA desaturase
SFA	Saturated fatty acids
TAA	Total amino acids
TPA	Texture profile analysis
UAA	Umami amino acids
UFA	Unsaturated fatty acids
